# Peroral cholangioscopy-assisted removal of a retained biliary fully covered metal stent using the inversion and traction technique

**DOI:** 10.1055/a-2479-1373

**Published:** 2024-12-10

**Authors:** Ivo Mendes, Francisco Vara-Luiz, Carolina Palma, Júlio Veloso, Jorge Fonseca, Pedro Pinto-Marques, Gonçalo Nunes

**Affiliations:** 1 70816 Gastroenterology, Hospital Garcia de Orta EPE, Almada, Portugal; 2 510440 Egas Moniz Center for Interdisciplinary Research, Egas Moniz School of Health and Science, Caparica, Portugal

A 45-year-old man with chronic pancreatitis was admitted with abdominal pain and jaundice caused by a benign biliary stricture. Endoscopic retrograde cholangiopancreatography (ERCP) confirmed a short, regular stricture in the distal common bile duct. A 60×10-mm fully covered self-expandable metal stent (FCSEMS) was placed for biliary drainage. After 6 months, a follow-up ERCP was performed, which revealed that the distal end of the FCSEMS was no longer visible in the lumen, as it had become entirely embedded in the duodenal wall due to hyperplastic tissue overgrowth. Multiple attempts to remove the stent using grasping forceps, a snare, and an extraction balloon were unsuccessful.

To induce tissue necrosis, a stent-in-stent technique was applied during three consecutive procedures performed over 2–4 weeks; however, all attempts to retrieve the FCSEMS failed. Consequently, a rescue ERCP with peroral cholangioscopy was scheduled to facilitate stent removal ( Video 1 ). During the procedure, hydrostatic balloon dilation improved the previous stent distortion caused by repeated manipulation. The cholangioscope (SpyGlass DS II; Boston Scientific, Boston, MA, USA) was advanced, confirming marked hyperplastic tissue overgrowth at the distal 2cm of the FCSEMS ( Fig. 1 ). Using SpyBite Max biopsy forceps, the proximal end of the stent was grasped and retracted, allowing partial inversion of the stent into its own lumen ( Fig. 2 ). This inversion maneuver facilitated grasping of the proximal end with foreign body biopsy forceps, introduced through the duodenoscope in a subsequent step. Moderate traction allowed complete inversion of the stent into the duodenal lumen, enabling successful removal of the FCSEMS without complications ( Fig. 3 , Fig. 4 ). Complete resolution of the biliary stricture was observed.

Successful removal of a retained biliary fully covered self-expandable metal stent (FCSEMS) using the cholangioscopy-assisted inversion and traction technique.Video 1

**Fig. 1:**
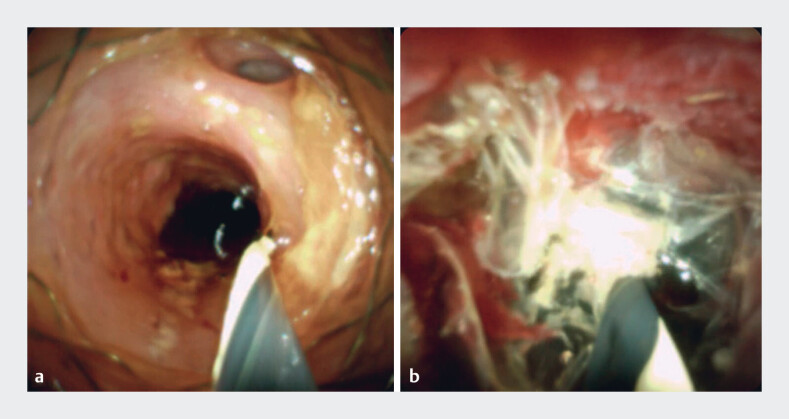
**a–b** Cholangioscopic view of the proximal end of the fully covered self-expandable metal stent (FCSEMS) ( **a** ) and marked hyperplastic tissue overgrowth on the distal end of the FCSEMS ( **b** ).

**Fig. 2:**
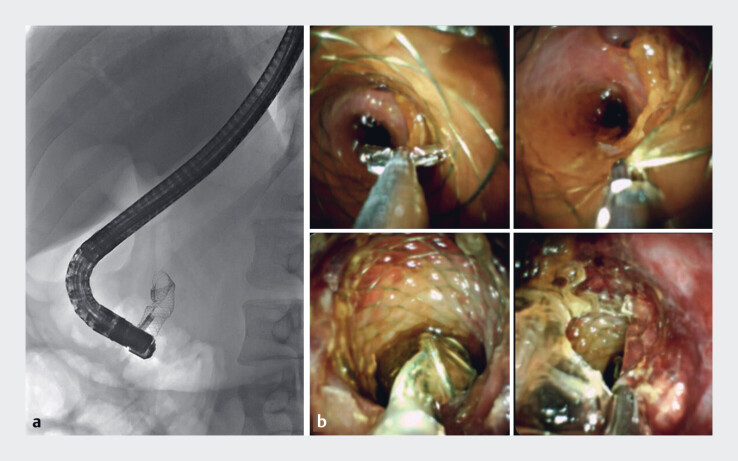
**a–b** Fluoroscopic ( **a** ) and cholangioscopic ( **b** ) images of partial inversion of the proximal end of the fully covered self-expandable metal stent (FCSEMS) using the SpyBite Max biopsy forceps.

**Fig. 3:**
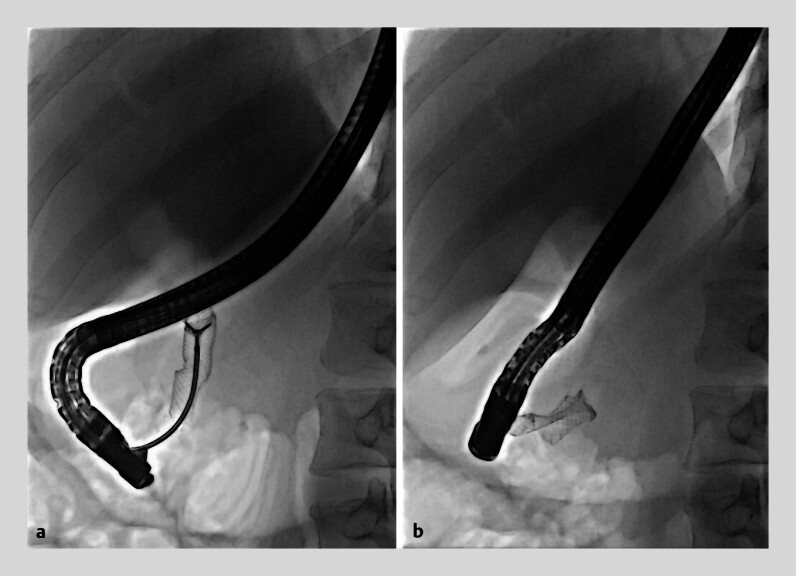
**a** Foreign body biopsy forceps grasping the inverted proximal end of the fully covered self-expandable metal stent. **b** Complete inversion of the stent into the duodenal lumen.

**Fig. 4:**
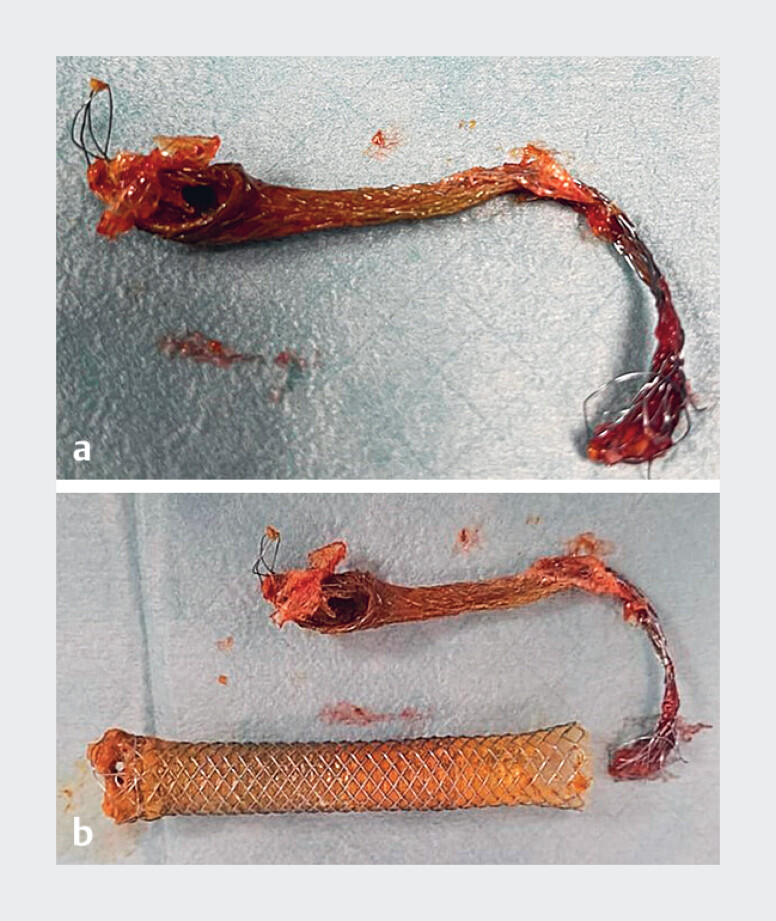
**a** A fully covered self-expandable metal stent (FCSEMS) removed using the peroral cholangioscopy-assisted inversion and traction technique. **b** The retained FCSEMS and the inner stent are shown.

Irretrievable biliary FCSEMSs typically result from proximal migration or hyperplastic tissue overgrowth. The use of peroral cholangioscopy is increasing in ERCP practice, with a growing range of clinical applications. Although the stent-in-stent technique has proven effective for removing retained biliary stents, this report presents a refractory case and describes an innovative, cholangioscopy-assisted approach to managing this challenging complication 1 2 3 4 .

Endoscopy_UCTN_Code_TTT_1AR_2AZ

## Notes

Endoscopy E-Videos https://eref.thieme.de/e-videos
*E-Videos* is an open access online section of the journal *Endoscopy* , reporting on interesting cases and new techniques in gastroenterological endoscopy. All papers include a high-quality video and are published with a Creative Commons CC-BY license. Endoscopy E-Videos qualify for HINARI discounts and waivers and eligibility is automatically checked during the submission process. We grant 100% waivers to articles whose corresponding authors are based in Group A countries and 50% waivers to those who are based in Group B countries as classified by Research4Life (see: https://www.research4life.org/access/eligibility/ ). This section has its own submission website at https://mc.manuscriptcentral.com/e-videos .
